# Supplementary material for: Natural phasic inhibition of dopamine neurons signals cognitive rigidity
Source: bioRxiv. 2024 Jul 13:2024.05.09.593320. Originally published 2024 May 9. Preprint. [Version 2] doi: 10.1101/2024.05.09.593320 (PMC11100816; doi:10.1101/2024.05.09.593320)
Supplement: Supplement 1 [file NIHPP2024.05.09.593320v2-supplement-1.pdf]

bioRxiv preprint doi: https://doi.org/10.1101/2024.05.09.593320; this version posted July 13, 2024. The copyright holder for this preprint (which was not certified by peer review) is the author/funder, who has granted bioRxiv a license to display the preprint in perpetuity. It is made available under aCC-BY 4.0 International license.

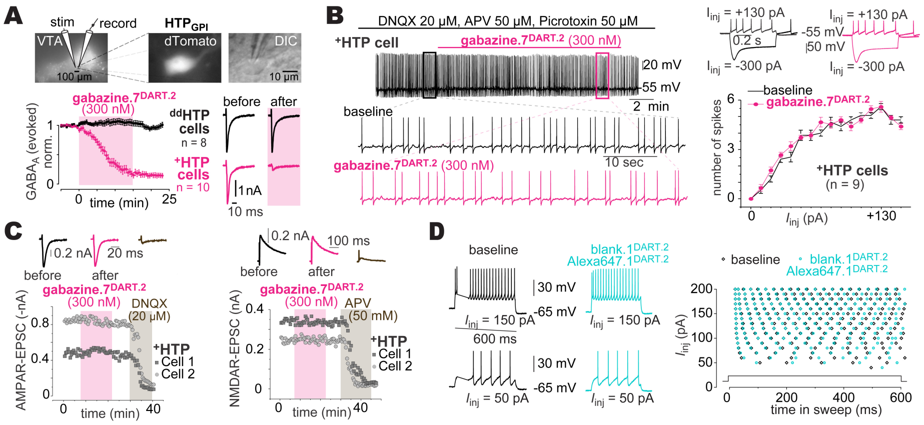

Fig. S1: Specificity of gabazine^{DART} to the GABA$_A$R

A: Gabazine.7$^{DART.2}$ validation. **Top:** evoked-IPSC configuration in VTA slice. **Bottom:** 300 nM gabazine.7$^{DART.2}$ has no impact on ^{dd}HTP neurons, while blocking IPSCs on $^{+}$HTP cells in under 15 min (86 ± 4% block). Data are mean ±SEM, cells normalized to baseline ($^{+}$HTP: n=10 cells; ^{dd}HTP: n=8 cells). Example traces to right.

B: Gabazine.7$^{DART.2}$ and VTA$_{DA}$ action potentials. **Left:** current clamp of a VTA$_{DA}$ neuron in the presence of picrotoxin (GABA$_A$ receptor blocker), DNQX (AMPA receptor blocker), and APV (NMDA receptor blocker). **Right:** quantification of action potential firing as a function of injected current; performed before (black) vs after (cyan) gabazine.7$^{DART.2}$ was tethered on each cell. Representative traces shown above. Error bars are mean ±SEM over cells (*n* = 9).

C: Gabazine.7$^{DART.2}$ and AMPARs/NMDARs. **Left:** 300 nM gabazine.7$^{DART.2}$ has no effect on $^{+}$HTP neuron AMPAR-EPSCs, which are subsequently blocked by 20 µM DNQX. Example traces above. **Right:** 300 nm gabazine.7$^{DART.2}$ has no effect on $^{+}$HTP neuron NMDAR-EPSCs, which are subsequently blocked by 50 µM APV. Example traces above.

D: Alexa647.1$^{DART.2}$ validation. Current clamp studies in VTA$_{DA}$ $^{+}$HTP neurons with 10:1 blank.1$^{DART.2}$ + Alexa647.1$^{DART.2}$. No significant change was observed before vs after Alexa647.1$^{DART.2}$ was tethered. Representative traces shown left.

bioRxiv preprint doi: https://doi.org/10.1101/2024.05.09.593320; this version posted July 13, 2024. The copyright holder for this preprint (which was not certified by peer review) is the author/funder, who has granted bioRxiv a license to display the preprint in perpetuity. It is made available under aCC-BY 4.0 International license.

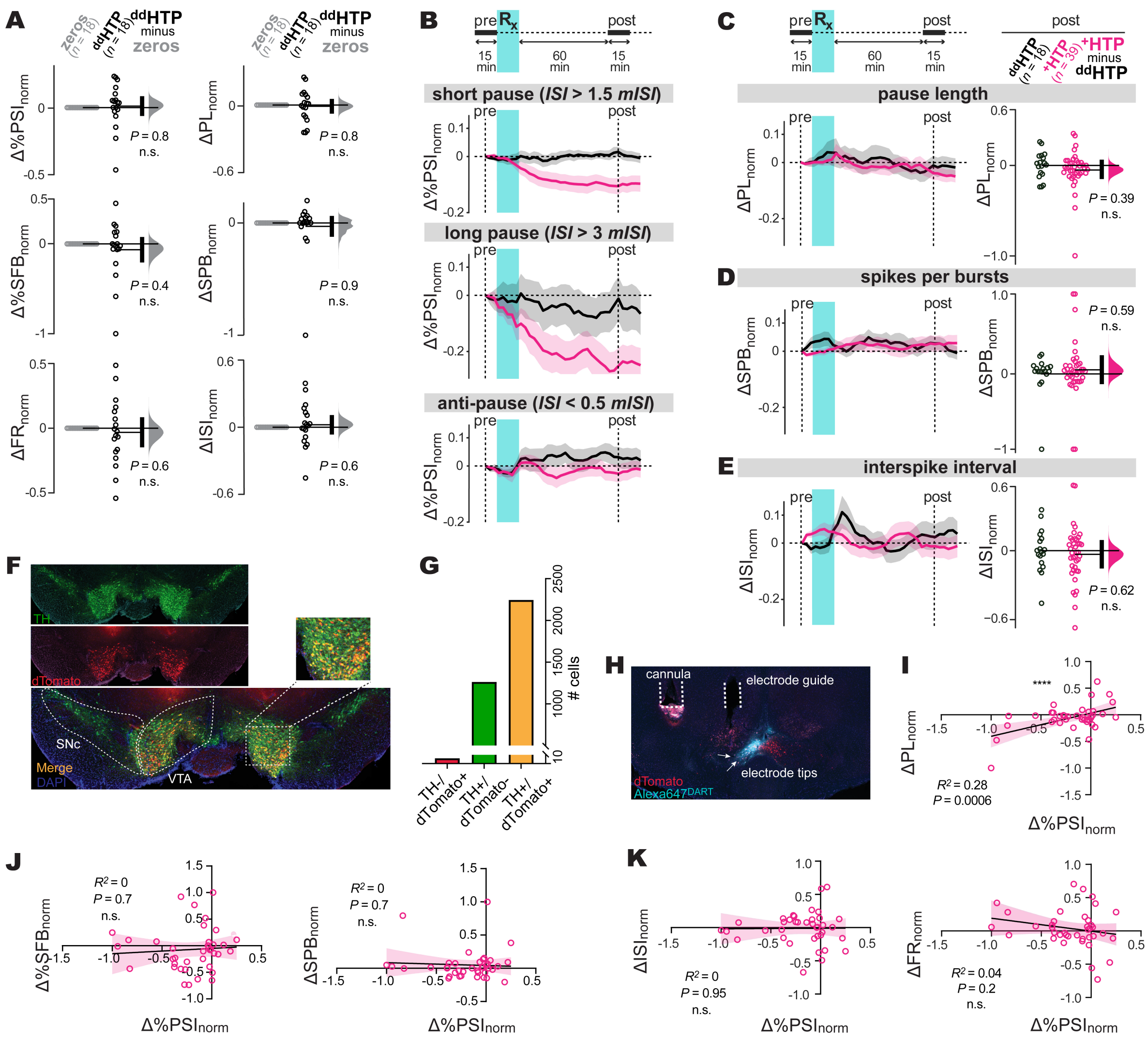

Fig. S2: Supporting data for *in vivo* electrophysiology

A: Control ^{dd}HTP metrics. Analysis of tonic, burst, and pause stability in ^{dd}HTP mice. Steady-state Δ_{norm} (1-hr post-gabazine^{DART}) with individual cells (circles), group means (thin horizontal lines), mean-difference bootstrap (grey distribution), and 95% CI of the two-sided permutation test (vertical black bar), comparing ^{dd}HTP cells to zero.

B: Examining robustness of pause result. Robustness analysis of our primary pause metric, ***%PSI***. The top two panels examine various "longer-than-average" metrics of pause occurrence (format as in **Fig. 1d**). The bottom panel examines a "shorter-than-average" metric to test for the asymmetry of effects.

C-E: Pause length, bursts, and ISI. Analysis of *PL* (pause length), *SPB* (spikes per burst), and *mISI* (median interspike interval); format as in Fig. 1d-f.

F-G: Cell counting. Representative histology and quantitative cell counting. Dopamine neurons (TH, tyrosine-hydroxylase, green). HTP expression (dTomato, red). Cell counting was performed from one representative brain. There were 2,244 double-labeled (dTomato$^+$/TH$^+$) cells. This represents 99.7% of all virus-positive cells (2,250 dTomato$^+$), and 64% of all dopaminergic cells (3,507 TH$^+$).

H: Electrode histology. Post-electrophysiology histology. HTP expression (dTomato, red); ligand capture (cyan); and electrode tips (arrows).

I: Pauses vs pause length. Correlation between *PL* (pause length) and ***%PSI*** from each $^+$HTP cell (circles, n=39), with regression ±95% CI (line and shading). Pearson's r^2 = 0.28, P = 0.0006.

J-K: Pauses vs other features. Correlation between all other metrics and ***%PSI***; format as above.

bioRxiv preprint doi: https://doi.org/10.1101/2024.05.09.593320; this version posted July 13, 2024. The copyright holder for this preprint (which was not certified by peer review) is the author/funder, who has granted bioRxiv a license to display the preprint in perpetuity. It is made available under aCC-BY 4.0 International license.

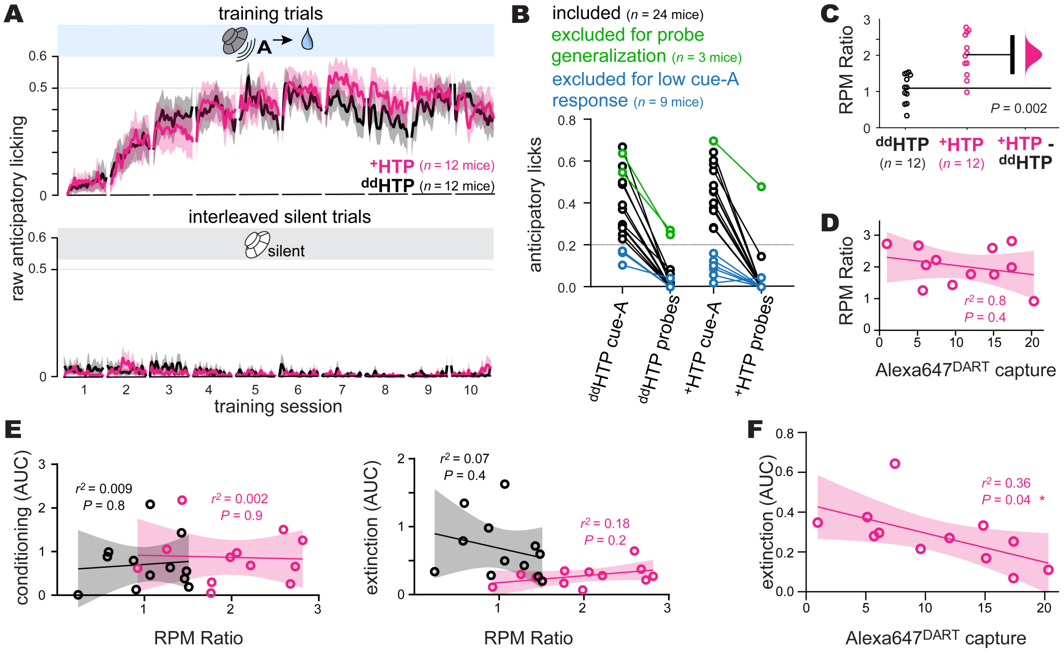

Fig. S3: Supporting data for Pavlovian extinction and conditioning assay

A: Training sessions. Lines and shading show anticipatory licking (fraction of time that beam is broken during the cue), mean ± SEM over mice (n = 12 ^{dd}HTP; n = 12 $^{+}$HTP). Both $^{+}$HTP and ^{dd}HTP mice develop robust anticipatory licking to cue A across training, while exhibiting little to no background licking during silent trials. Note that this figure shows raw (non-normalized) anticipatory licking, whereas the main-text figures display normalized anticipatory licking, with day-10 anticipatory as a constant of normalization for each animal.

B: Behavioral inclusion criteria. We required robust anticipatory licking to cue A (raw anticipatory licking greater than 0.2) and low responsiveness to cue B probes (less than 30% of cue A anticipatory licking). A total of 9 mice (3 ^{dd}HTP and 6 $^{+}$HTP) were excluded for lack of cue A responsiveness (blue). Of the remaining 27 mice, only 3 mice (2 ^{dd}HTP and 1 $^{+}$HTP) were excluded for probe generalization (green). Thus 89% (24 of 27) successfully discriminated cue B.

C: Locomotion. Ratio of the average treadmill RPM post-gabazine^{DART} (day 11-12) divided by pre-gabazineDART (day 8-10). Individual mice (circles), group means (thin horizontal lines), mean-difference bootstrap (pink distribution), and 95% CI of the two-sided permutation test (vertical black bar). As previously reported (*43*), disinhibition of VTA$_{DA}$ neurons enhances locomotion.

D: Locomotion vs histology. Correlation between RPM ratio and Alexa647^{DART} capture in the dorsal VTA of $^{+}$HTP mice (*n*=12). Mice (circles), regression ±95% CI (line and shading). Pearson's r^2 = 0.08, *P* = 0.4 indicates no significant correlation.

E: Locomotion vs reward learning. Correlation between RPM ratio and our two measures of reward learning: conditioning AUC (left) and extinction AUC (right). Mice (circles; *n*=12 ^{dd}HTP; *n*=12 $^{+}$HTP) and regression ±95% CI (line and shading). Pearson's tests show no significant correlation (r^2 and *P* values as indicated).

F: Extinction learning vs histology. Correlation between extinction (AUC) and Alexa647^{DART} capture in the dorsal VTA of $^{+}$HTP mice (*n*=12). Mice (circles), regression ±95% CI (line and shading). Pearson's r^2 = 0.36, *P* = 0.04 indicates a significant correlation, with higher levels of target engagement corresponding to faster rates of extinction.

bioRxiv preprint doi: https://doi.org/10.1101/2024.05.09.593320; this version posted July 13, 2024. The copyright holder for this preprint (which was not certified by peer review) is the author/funder, who has granted bioRxiv a license to display the preprint in perpetuity. It is made available under aCC-BY 4.0 International license.

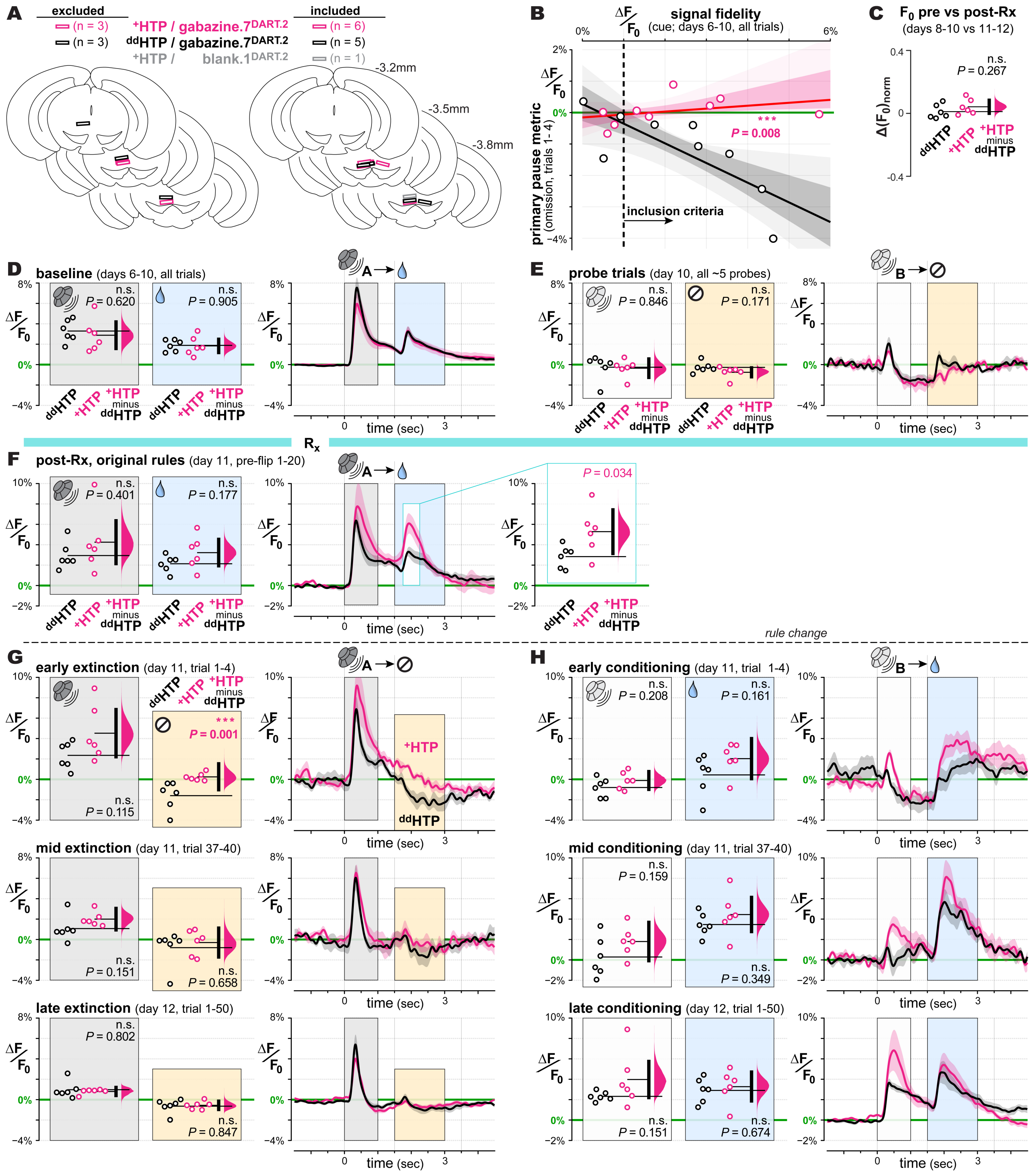

(legend on next page)

bioRxiv preprint doi: https://doi.org/10.1101/2024.05.09.593320; this version posted July 13, 2024. The copyright holder for this preprint (which was not certified by peer review) is the author/funder, who has granted bioRxiv a license to display the preprint in perpetuity. It is made available under aCC-BY 4.0 International license.

Fig. S4: Supporting data for fiber photometry studies

A: Fiber placement. Optic fibers were placed dorsal to the VTA and equivalently spread between control and experimental conditions. 6 mice (3 $^{+}$HTP/gabazine^{DART} and 3 ^{dd}HTP/gabazine^{DART}) were excluded based on GCaMP8f signal levels (left). 12 mice (6 $^{+}$HTP/gabazine^{DART}, 5 ^{dd}HTP/gabazine^{DART}, and 1 $^{+}$HTP/blank^{DART}) were included in further analysis (right).

B: Photometry inclusion criteria. Signal-fidelity metric (cue-evoked burst, days 6-10) reflects GCaMP8f expression and its coupling efficiency to the fiber-optic. This pre-gabazine^{DART} signal-fidelity metric is plotted against our main post-gabazine^{DART} pause metric (omission pause, day 11 extinction trials 1-4). Data from individual mice (circles), regression fits (lines), and regression 95% and 68% CI (light and dark shading) are shown for ^{dd}HTP (black, n = 9) and $^{+}$HTP (pink, n = 9) mice. With all data included, there is a clear statistical difference between ^{dd}HTP and $^{+}$HTP mice (two-sided permutation slope test, P = 0.008). Mice above a signal-fidelity threshold (to the right of the dashed line at 1% $\Delta F/F_0$) are included in the subsequent analyses.

C: Tonic activity. Changes in baseline GCaMP8f intensity on days 8-10 ($F_{0,pre}$) vs days 11-12 ($F_{0,post}$) are analyzed according to $\Delta(F_0)_{norm} = (F_{0,post} - F_{0,pre}) / (F_{0,post} + F_{0,pre})$. Individual mice (circles), group means (thin horizontal lines), mean-difference bootstrap (pink distribution), and 95% CI of the two-sided permutation test (vertical black bar); $^{+}$HTP and ^{dd}HTP cells do not differ significantly (P=0.267).

D: Days 6-10 rewarded cue A trials. Right: GCaMP8f responses in ^{dd}HTP (black) vs $^{+}$HTP (pink) mice during days 6-10 (average over all cue A trials). Right panel shows the time course of $\Delta F/F_0$ mean ± SEM over mice (n = 6 ^{dd}HTP mice; n = 6 $^{+}$HTP mice). Analysis of $\Delta F/F_0$ during cue (gray) and reward (yellow) is plotted in the left panel of corresponding color. **Left:** individual mice (circles), group means (thin horizontal lines), mean-difference bootstrap (pink distribution), and 95% CI of the two-sided permutation test (vertical black bar). $^{+}$HTP and ^{dd}HTP mice were not statistically different during cue (0 – 1 sec interval; P=0.620) or reward (1.5 – 3 sec interval, P=0.905). Examination of an additional, narrow-time reward interval (1.75 – 2.25 sec) was also not significant (P=0.968; not shown).

E: Day 10 unrewarded cue B probes. Format as in panel **d**; for pre-gabazine^{DART} unrewarded cue B probes. Cue-evoked signals (0 - 1 sec) were not statistically different in $^{+}$HTP vs ^{dd}HTP mice (P=0.846). Signals in the unrewarded interval displayed a non-significant trend (1.5 - 3 sec; P=0.171), which remained non-significant over a narrow interval (1.75 – 2.25 sec; P=0.054; not shown).

F: Day 11 rewarded cue A trials. Format as in panel **d**; post-gabazine^{DART} rewarded cue A trials (prior to rule change). Cue-evoked signals (0 - 1 sec) were not statistically different in $^{+}$HTP vs ^{dd}HTP mice (P=0.401). For reward-evoked signals, $^{+}$HTP mice exhibited a trending increase (1.5 - 3 sec; P=0.117) which was weakly significant within a narrow interval (1.75 – 2.25 sec; P=0.034, right inset).

G: Days 11-12 cue A extinction. Format as in panel **d**; post-gabazine^{DART} cue A extinction (early, middle, and late trials from top to bottom). Cue-evoked signals (0 - 1 sec) showed a non-significant trend during early (P=0.115) and middle (P=0.151) but not late (P=0.802) trials. Omission-pause signals (1.5 - 3 sec) were evident in ^{dd}HTP mice yet absent in $^{+}$HTP mice during early trials (P=0.001). This difference was not apparent during middle (P=0.658) and late (P=0.847) trials owing to the lack of pauses in control mice. Examination of a narrow interval (1.75 – 2.25 sec) upheld these results (early P=0.00045; middle P=0.448; late P=0.945).

H: Days 11-12 cue B conditioning. Format as in panel **d**; post-gabazine^{DART} cue B conditioning (early, middle, and late trials from top to bottom). Cue-evoked signals (0 - 1 sec) showed a non-significant trend (early P=0.208; middle P=0.159; late P=0.151). Reward-evoked signals displayed a similar trend for both the full (1.5 - 3 sec) interval (early P=0.161; middle P=0.349; late P=0.674) and narrow (1.75 – 2.25 sec) interval (early P=0.123; middle P=0.306; late P=0.640).

bioRxiv preprint doi: https://doi.org/10.1101/2024.05.09.593320; this version posted July 13, 2024. The copyright holder for this preprint (which was not certified by peer review) is the author/funder, who has granted bioRxiv a license to display the preprint in perpetuity. It is made available under aCC-BY 4.0 International license.

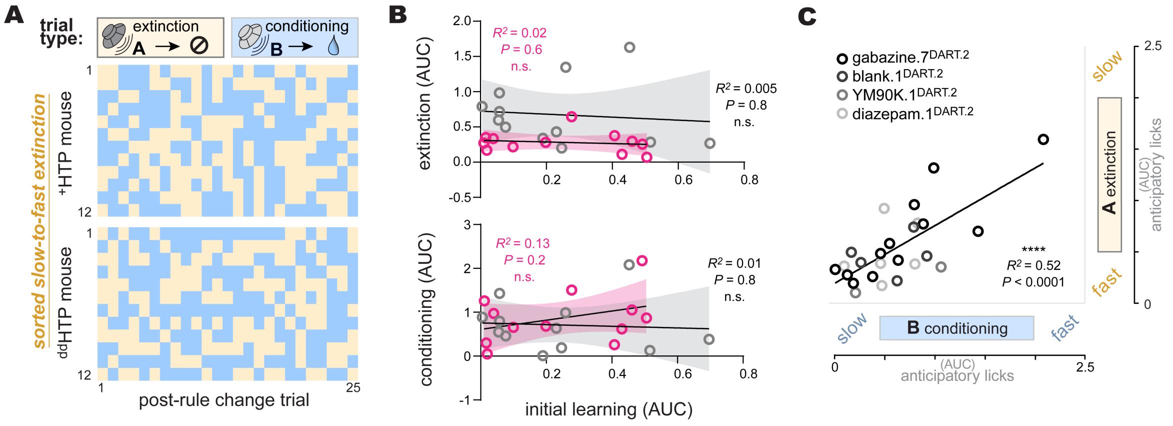

Fig. S5: Supporting data for within-mouse behavioral correlations

A: Visual inspection of random order of interleaved trials. Each row is one mouse, sorted by extinction from the slowest to fastest (top to bottom). Columns indicate the first 25 trials after the rule-change, with trial type indicated by color (yellow = cue A extinction, blue = cue B conditioning). No visually discernable pattern is apparent in either $^{+}$HTP mice (n=12) or ^{dd}HTP mice (n=12).

B. Initial training vs later reward learning. Correlation between initial learning rates (AUC over training days 1-2) and our two measures of reward learning: extinction AUC (top) and conditioning AUC (bottom). Mice (circles; n=12 ^{dd}HTP; n=12 $^{+}$HTP) and regression ±95% CI (line and shading). Pearson's tests show no significant correlation (r^2 and P values as indicated).

C: Phenotypic spectrum across pooled controls. Conditioning-*AUC* vs extinction-*AUC* measured within-mouse. Individual mice (circles), regression fit (line), are shown for mice pooled from ongoing control experiments. All data are from ^{dd}HTP mice infused with various ligands, including gabazine.7$^{DART.2}$, blank.1$^{DART.2}$, YM90K.1$^{DART.2}$, or diazepam.1$^{DART.2}$. Pearson's r^2=0.52, P<0.0001. 75

bioRxiv preprint doi: https://doi.org/10.1101/2024.05.09.593320; this version posted July 13, 2024. The copyright holder for this preprint (which was not certified by peer review) is the author/funder, who has granted bioRxiv a license to display the preprint in perpetuity. It is made available under aCC-BY 4.0 International license.

Table S1: Key Resources

REAGENT or RESOURCE	SOURCE	IDENTIFIER
**Bacterial and virus strains**		
AAV$_{rh10}$-CAG-DIO-$^{+}$HTP$_{GPI}$-2A-dTomato-WPRE	Duke Viral Vector Core<br>VectorBuilder	AAV10-X0117A, Lot 200807, 210215, 220331, VB220331
AAV$_{rh10}$-CAG-DIO-^{dd}HTP$_{GPI}$-2A-dTomato-WPRE	Duke Viral Vector Core<br>VectorBuilder	AAV10-M6360D<br>Lot 200203, 210420, VB220331
AAV$_{rh10}$-CAG-CreON-W3SL-$^{+}$HTP$_{GPI}$-IRES-dTomato-Farnesylated	Duke Viral Vector Core<br>VectorBuilder	AAV10-6771A,<br>Lot 220328, VB220209
AAV$_{rh10}$-CAG-CreON-W3SL-^{dd}HTP$_{GPI}$-IRES-dTomato-Farnesylated	Duke Viral Vector Core<br>VectorBuilder	AAV10-6829B,<br>Lot 220328, VB220209
AAV9-CAG-FLEX-jGCaMP8f-WPRE	Addgene	RRID: 162382
**Chemicals, peptides, and recombinant proteins**		
gabazine.7$^{DART.2}$	Shields et al 2023	Lot 201109, 220512
Alexa647.1$^{DART.2}$	Shields et al 2023	Lot 200213
blank.1$^{DART.2}$	Shields et al 2023	Lot 210418
YM90K.1$^{DART.2}$	Shields et al 2023	Lot 180712c, 200725
diazepam.1$^{DART.2}$	Shields et al 2023	Lot 210628
Experimental models: Organisms/strains		
B6.SJL-Slc6a3tm1.1(cre)Bkmn/J	Jackson Labs	RRID:IMSR_JAX:006660
**Software and algorithms**		
MATLAB	MathWorks, Inc	Version 2017b, 2018a, 2020b
OpenEphys	Siegle et al 2017	Version 5.5.3, 6
Spyking Circus	Yger et al 2018	Version 1.0.1
Synapse	Tucker-Davis Technologies	Version 89-51248
Prism	GraphPad	Version 9.5.1, 10.0.3
ilastik	Berg et al 2019	Version 1.4.0.post1

bioRxiv preprint doi: https://doi.org/10.1101/2024.05.09.593320; this version posted July 13, 2024. The copyright holder for this preprint (which was not certified by peer review) is the author/funder, who has granted bioRxiv a license to display the preprint in perpetuity. It is made available under aCC-BY 4.0 International license.

Table S2. Detailed Author Contributions

Name	ORCID	Contribution
Sasha C.V. Burwell	0000-0003-3553-1365	**Fig. 1, Fig. S2:** Conceived, designed, and performed all *in vivo* electrophysiology experiments. Optimized surgical and infusion procedures for dual electrode recording and DART manipulation of VTA dopamine neurons. Performed all spike-sorting analysis to ensure consistent extraction of putative dopamine neurons. Wrote software to extract and analyze sorted cell templates. Performed statistical analysis of spiking data. Performed histology (sample preparation, imaging, cell counting). **Fig. 2, 4, Fig. S3, 5:** Conceived, designed, and performed all behavior experiments. Designed and built the reward-learning assay. Wrote software to run the assay and to collect and analyze data. Performed statistical analysis of behavior data. Optimized AAV serotype, promoter, surgical procedure, and infusion procedure for utilizing DART in VTA dopamine neurons. Performed all histology (sample preparation, imaging, image segmentation, and image analysis). **Fig. 3, Fig. S4:** Conceived, designed, and performed all fiber photometry experiments. Optimized AAV, surgical procedure, and infusion procedure for dual GCaMP recording and DART manipulation of VTA dopamine neurons. Wrote software for extracting and calculating dF/F signals. Performed statistical analysis of fiber photometry data. Performed all histology (sample preparation, imaging, and image segmentation). **Fig. S1:** Performed virus injection surgeries for slice electrophysiology experiments. Wrote original manuscript draft and prepared original versions of **Fig. 1-4** and **Fig. S2-5**. Edited and revised paper and figures. Managed and curated data, including managing deposition into repositories and preparing protocols, and scheduled/planned all experiments.<br><br>**CRediT**: Conceptualization, Methodology, Software, Validation, Formal Analysis, Investigation, Data Curation, Writing - Original Draft, Writing - Review & Editing, Visualization, Project Administration
Haidun Yan	0000-0003-0916-8865	**Fig. S1:** Conceived, designed, and performed all electrophysiology experiments in VTA brain slices. Performed GABAR-mediated eIPSCs recording by voltage clamp and manipulated gabazine.7$^{DART.2}$ effects from $^{+}$HTP or ^{dd}HTP virus injected VTA DA neurons. Performed spontaneous and evoked action potentials recording by current-clamp, evaluated which gabazine.7$^{DART.2}$ effects on membrane excitability and endogenous channels from $^{+}$HTP virus injected VTA DA neurons. Performed AMPAR- and NMDAR-mediated eEPSCs recording by voltage clamp and evaluated gabazine.7$^{DART.2}$ effects on excitatory synaptic function in $^{+}$HTP positive VTA DA neurons. Performed evoked action potentials recording in $^{+}$HTP positive VTA DA neurons, tested the effects of blank.1$^{DART.2}$ + Alexa647.1$^{DART.2}$.<br>Wrote original draft of methods for electrophysiology in brain slices, and prepared original version of **Fig. S1.** Reviewed and provided feedback on manuscript.<br><br>**CRediT**: Methodology, Validation, Formal Analysis, Investigation, Resources, Writing - Review & Editing, Visualization

bioRxiv preprint doi: https://doi.org/10.1101/2024.05.09.593320; this version posted July 13, 2024. The copyright holder for this preprint (which was not certified by peer review) is the author/funder, who has granted bioRxiv a license to display the preprint in perpetuity. It is made available under aCC-BY 4.0 International license.

Shaun S.X Lim	0000-0001-9312-6275	Designed and cloned different variants of the HTP plasmid constructs, including the optimized variant in the experiment. Assisted with validating HTP expression and HTL capture in dopamine neurons. Separately validated locomotor effects from tethering gabazine.7$^{DART.2}$ on VTA dopamine neurons. Reviewed and provided feedback on manuscript.<br><br>**CRediT**: Methodology, Validation, Resources, Writing - Review & Editing
Brenda C. Shields	0000-0001-9036-2686	Assisted in original development and characterization of DART reagents. Performed cloning and validation of viral constructs. Prepared all DART and virus aliquots.<br><br>**CRediT**: Methodology, Validation, Resources, Writing - Review & Editing, Project Administration
Michael R. Tadross	0000-0002-7752-6380	Senior Author & Lead Contact.<br><br>**CRediT**: Conceptualization, Methodology, Software, Validation, Formal Analysis, Resources, Writing Review & Editing, Visualization, Supervision, Project Administration, Funding Acquisition (Duke Startup. NIH: 1RF1MH117055, 1DP2MH1194025)
